# Anti-Diabetic Activity of Bioactive Compound Extracted from *Spondias mangifera* Fruit: In-Vitro and Molecular Docking Approaches

**DOI:** 10.3390/plants11040562

**Published:** 2022-02-21

**Authors:** Mohammad Khalid, Mohammed H. Alqarni, Abdulrhman Alsayari, Ahmed I. Foudah, Tariq M. Aljarba, Mohammad Mukim, Mubarak A. Alamri, Shahabe Saquib Abullais, Shadma Wahab

**Affiliations:** 1 Department of Pharmacognosy, College of Pharmacy, Prince Sattam Bin Abdulaziz University, P.O. Box 173, Al-Kharj 11942, Saudi Arabia; m.alqarni@psau.edu.sa (M.H.A.); a.foudah@psau.edu.sa (A.I.F.); t.aljarba@psau.edu.sa (T.M.A.); 2 Department of Pharmacognosy, College of Pharmacy, King Khalid University, Abha 61421, Saudi Arabia; alsayari@kku.edu.sa (A.A.); shad.nnp@gmail.com (S.W.); 3 Department of Pharmacology, Kota College of Pharmacy, Kota 324005, Rajasthan, India; mukim.life@gmail.com; 4 Department of Pharmaceutical Chemistry, College of Pharmacy, Prince Sattam Bin Abdulaziz University, Al-Kharj 11942, Saudi Arabia; mubarak@psau.edu.sa; 5 Department of Periodontics and Community Dental Sciences, College of Dentistry, King Khalid University, Abha 61421, Saudi Arabia; drsaquib24@gmail.com

**Keywords:** *Spondias mangifera*, antidiabetic, antioxidant, in vitro, pharmacokinetic, molecular docking

## Abstract

*Spondias mangifera* is a drupaceous fruit popular for its flavour and health advantages. There is little scientific knowledge about *S. mangifera*, despite its widespread usage in traditional medicine, in the North-Eastern region of India. Inhibiting the key carbohydrate hydrolysing enzymes is one of the strategies for managing diabetes. Therefore, this study studied the antioxidant and anti-diabetic properties of different fraction *S. mangifera* fruit extract (SMFFs) from Indian geographical origin by in vitro experimental assays and *silico* docking simulation studies. The ADMET prediction for active substances was also investigated using the AdmetSAR database. Based on the binding affinity/molecular interactions between phytocompounds and target enzymes, in silico investigations were done to confirm the in vitro enzymatic inhibitory capability. β-sitosterol in EtOH-F was analysed using RP-HPLC with RP-C18 column as stationary phase and photo diode array detector. The percentage of β-sitosterol was found to be 1.21% ± 0.17% of total weight of extract (*w/w*). *S. mangifera* fruit ethanolic extract had a significant inhibitory concentration of 50% against free radicals produced by ABTS (89.71 ± 2.73%) and lipid peroxidation assay (88.26 ± 2.17%) tests. Similarly, the in vitro antidiabetic test findings indicated that *S. mangifera* inhibited alpha-amylase (73.42 ± 2.01%) and alpha-glucosidase (79.23 ± 1.98%) enzymes dose-dependently. The maximum glycosylated Hb percentage inhibitory activity shown in the ethanolic fraction was (83.97 ± 2.88%) at 500 µg/mL. The glucose uptake of the ethanolic fraction by the yeast cell showed significant (*p* < 0.05) at 500 µg/mL when compared with metformin (91.37 ± 1.59%), whereas the other fraction did not show the uptake of glucose by the yeast cell at the same concentration. In the docking study, the main phytoconstituents of *S. mangifera* fruit, such as oleanolic acid, beta-sitosterol, and beta amyrin, show strong affinity for pancreatic α-amylase. These results imply that *S. mangifera* has α-amylase and α-glucosidase inhibitory properties and may be used as antidiabetic with antioxidant characteristics.

## 1. Introduction

Diabetes mellitus (DM) is a chronic endocrine system condition that results in hyperglycemia. It is characterized by long-term chronic hyperglycemia, which eventually leads to ultimate organ failure. DM has become a worldwide public health crisis affecting the lifestyles of the majority world population [1]. DM is expected to impact 783 million people in the world by 2045, up from 537 million in 2021. It is one of the most severe health issues of the twenty-first century, according to the American Diabetes Association, and continues to be one of the fatal illnesses [2,3]. The rising prevalence of diabetes is due to several circumstances, such as oxidative stress caused by the free radical generation that may cause β-cells in the pancreas to malfunction, decreased glucose tolerance, and insulin resistance. α-Amylase and α-glucosidase are two crucial key enzymes that break down carbohydrates and support the body to absorb them in the intestines [4].

Antioxidants are a group of molecules that protect against ROS species in living things by competing with other molecules that are important for ROS oxidation [5]. Antioxidants are preventive therapy that may help lessen the consequences linked with oxidative stress [6]. Antioxidants are regarded useful therapeutic alternatives since oxidative stress involves a significant aspect in the development of diabetes mellitus. In addition, studies have shown that antioxidants and α-glucosidase inhibitors from natural sources, like fruits, may have anti-diabetic benefits, so these natural compounds have been getting a lot of attention from scientists around the world [7]. Phytosterols are abundant in plants, vegetables, fruits, and nuts. β-sitosterol is the most common phytosterol, and several in-vitro and in-vivo investigations have shown that it has a variety of biological effects. The antioxidant function of β-sitosterol has been shown in several scientific investigations to work as a moderate radical scavenger chemically, as well as a membrane stabilizer physically [8,9].

*Spondias mangifera* of the family Anacardiaceae is a glabrous tree. It grows to 10.5 m tall and has smooth ash-colored bark with a straight trunk and a distinct woody scent [10]. The bark is used ethnopharmacologically as a tonic, a refrigerant, cure muscular and articular rheumatism, dysentery, and diarrhoea [11]. Indian states, Punjab, West Bengal, Maharashtra, Assam, and Orissa, are the main places where this is cultivated as edible fruits [12]. The leaves are aromatic, astringent, acidic, and are used to flavour food. While the juice is used to treat earaches [13,14]. Sitosterol, stigmast-4-en-3-one, daucosterol, 24-methylene, lignoceric acid, and cycloartanone are found in the aerial sections of the plant. Other substances found in the fruits include β-amyrin and oleanolic acid [15]. This plant’s ripe fruit juice is high in vitamins and can be employed as a nutraceutical agent [16]. The root bark powder of the plant has been indicated for menstrual management, as well as anticancer, antihistamine, antipyretic, and antispasmodic properties [17,18,19,20,21]. Additionally, the plant’s fruits and barks treat diabetes [16]. In rats, methanolic extracts of stem heartwood had shown hepatoprotective action against carbon tetrachloride-induced liver toxicity [22].

Phytomedicines derived from herbs and shrubs have been used in traditional healing procedures all across the globe since antiquity [23]. Plant-based drugs or herbal compositions may play a key role in managing DM [24]. Synthetic oral hypoglycemic medicines (Meglitinides, sulfonylureas, glitazones, gliptins, sulfonylureas, biguanides) and other currently available antidiabetic treatments have many side effects, such as sleepiness, anorexia, stomach pain, weight gain, etc. [25]. Therefore, a comprehensive scientific examination of traditionally beneficial medicinal plants is required to investigate their antidiabetic efficacy using current experimental instruments and procedures. Computational molecular modelling has emerged as a significant sector in the natural product drug development process. In silico drug design tools enhance the discovery of novel medications from natural products. Computational modelling sheds light on the molecular recognition processes underlying the interaction between disease-related target macromolecules with naturally occurring drug-like substances [26].

There is little scientific knowledge about *S. mangifera*, despite its widespread usage in traditional medicine, in the North-Eastern region of India. Therefore, this study examined the antioxidant and anti-diabetic properties of different fractions of *Spondias mangifera* fruit extract from Indian geographical origin. Furthermore, in silico using docking simulations experiments were conducted to confirm the in vitro enzymatic inhibitory capacity of phytocompounds, which was determined by the binding affinity and molecular couplings between phytochemicals and target enzymes. ADMET prediction studies were conducted to acquire a better knowledge of the association between activity and drug potency.

## 2. Material and Methods

### 2.1. Collection of Plant Materials and Authentication

The fruits of *S. mangifera* were collected from a local market in Lucknow, India. The fruits were authenticated by a taxonomist at the Department of Pharmacognosy, Faculty of Pharmacy, Integral University, Lucknow, India. For future reference, a voucher specimen (IU/PHAR/HRB/20/16) was submitted to the herbarium.

### 2.2. Chemicals

In this investigation, all of the chemicals utilized were of the analytical grade, and they were all obtained from Sigma Aldrich (St. Louis, MO, USA).

### 2.3. Extraction and Fractionation

The *S. mangifera* fruits (including peels) were collected, washed, and sliced into small pieces. Fruit pieces were spread out on a dry open area at room temperature for air drying. Further fruit pieces were dried in an oven at a regulated temperature of 40 °C until a consistent weight was reached. Afterward, the dried fruit pieces were ground into a coarse powder. They were then sieved and stored for future use. The *S. mangifera* fruits powder was defatted first with petroleum ether followed by the maceration with the methanol for the 3 days with occasional stirring. The extract was filtered using Whatman filter paper and evaporated at a low temperature and pressure on a rotatory evaporator to get a viscous and sticky mass. Fractionation of methanolic crude extract was done using hexane (Hx-F), chloroform (Ch-F), and ethanol (EtOH-F) solvents as per increasing order of solvent polarity. Each fraction was dried at 40 °C using a rotary evaporator. After drying, *S. mangifera* fruits fractionations (SMFFs) were stored at 4 °C until further usage [16].

### 2.4. HPLC Analysis of β-Sitosterol

The RP-HPLC system with a PDA detector (e2695 Separation module, Waters, Milford, MA, USA) was used for the analysis. The analysis was performed using an HPLC system heated to 30 °C and equipped with RP-C18 column (100 × 2.0 mm, 3.0 mm, Merck, Japan) as stationary phase. The solutions 10 μL each of ethanolic fraction and standards β-sitosterol were injected in the automated HPLC system. The whole separation was accomplished in isocratic mobile phase using acetonitrile: methanol in the ratio (80:20) with a flow rate of 1.0 mL min^−1^ and the column was set at ambient temperature. The sample and standard were detected at 212 nm. The presence of β-sitosterol were identified by comparing standard retention time (Rt) with extract [27].

### 2.5. In-Vitro Scavenging Activity

#### 2.5.1. Effect of SMFFs on ABTS Antioxidant Activity

7 mM ABTS solution was combined with 2.45 mM potassium persulfate in water to form the ABTS radical (1:1) ratio and maintained in a dark room for 24–48 h. Then, methanol was added to dilute the mixture to a final absorbance of 0.7 at 734 nm. Then 2.94 mL of the ABTS solution was combined with 60 mL of each extract and incubated in the dark for 20 min at 37 °C. The absorbance was determined at 734 nm after incubation. The below equation was used to figure out the percentage of inhibition [28].

% inhibition = [A_0_ − A_1_/A_0_] × 100

The absorbance values of the control (Blank) and the test sample, A_0_ and A_1_, are shown in the following equation.

#### 2.5.2. Effect of SMFFs on Percentage Inhibition of Lipid Peroxidation

The lipid peroxide produced was quantified using a modified thiobarbituric acid-reactive species (TBARS) test employing egg yolk homogenates as the lipid-rich medium. Malondialdehyde (MDA), a by-product of polyunsaturated fatty acid oxidation, interacts with two molecules of thiobarbituric acid (TBA) to form a pinkish-red chromogen with a maximum absorbance at 532 nm. In a test tube, egg homogenate (250 µL, 10% in distilled water, *v/v*) and 50 µL of extract were combined, and the volume was increased to 500 µL with distilled water. Then, 25 µL FeSO4 (0.07 M) was added to the above mixture and incubated for 30 min to produce lipid peroxidation. Following that, 750 µL of 20% acetic acid (pH 3.5), 750 µL of 0.8 percent TBA (*w/v*) (made in 1.1 percent sodium dodecyl sulphate), and 25 µL of 20% TCA were added, mixed thoroughly, and heated for 60 min in a boiling water bath. The mixture was cooled, then to each tube, 3.0 mL of 1-butanol was filled and centrifuged for 10 min at 3000 rpm. At 532 nm, the absorbance of the organic top layer was measured in comparison to 3 mL butanol. Instead of extract, 50 µL of distilled water was utilised as a blank [29].

### 2.6. In-Vitro Antidiabetic Activity

#### 2.6.1. Effect of SMFFs on α-Amylase Inhibitory Activity

The inhibitory impacts of extract fractions on alpha-amylase were determined employing the modified methodology reported by Bhutkar and Bhise (2012) [29]. The inhibition of α-amylase was determined by measuring the amount of reducing sugar (maltose equivalent) released during the experiment. The α-amylase enzyme inhibitory activity was measured as a reduction in the number of units of maltose released. The maltose equivalent was calculated using a modified dinitro salicylic acid (DNS) procedure. First, 1 mL of the extracts were pre-incubated for 30 min with α -amylase 1 U/mL; then, 1 mL of a 1% *w/v* starch solution was added. The mixture was further incubated at 37 °C for an additional 10 min. The reaction was then halted with the addition of 1 mL DNS reagent (12.0 g sodium potassium tartrate tetrahydrate in 8 mL of 2 M NaOH and 96 mM 3,5-dinitrosalicylic acid solution). The contents were heated for 5 min in a boiling water bath. A blank was made without amylase and a second without plant extracts, both of which were substituted with equal amounts of buffer (20 mM Sodium phosphate buffer with 6.7 mM Sodium chloride, pH 6.9 at 20 °C). At a wavelength of 540 nm, the absorbance was determined. A standard graph was used to determine the reducing sugar released by starch as maltose equivalent, and acarbose was utilized as a positive control. The extracts were diluted in the buffer to achieve final concentrations of 5 mg/mL, 7 mg/mL, and 9 mg/mL. The anti-diabetic activity was measured by the % inhibition of α-amylase, which was computed using the following equations:

% reaction = (maltose) test/(maltose) control × 100% inhibition = 100% reaction

#### 2.6.2. Effect of SMFFs on α-Glycosidase Inhibitory Assay

Monteiro de Souza et al. method was used to examine the inhibitory activity of *S. Mangifera* extracts for α-glucosidase [30]. The plant samples were incubated with 1 U/mL alpha-glucoside enzyme for 5 min after being diluted to 200 µL. It took 30 min at 37 degrees Celsius to start the reaction after 200 µL of the material reagent (pNpG, 3 mM produced in 10 mM of phosphate buffer pH 6.9) was added. The process was stopped by adding sodium molecules at a concentration of 100 mM, and the response was measured at an absorbance of 405 nm. Blanks were made by substituting enzyme with distilled water. Following these steps, the percentage of α-glucosidase inhibition was determined:

Percentage inhibition = (absorption of control − absorption of test) 100/absorption of control

#### 2.6.3. Effect of SMFFs on Non-Enzymatic Glycosylation of Haemoglobin Assay

To determine the anti-diabetic effectiveness of *S. mangifera*, colorimetric measurements were used at 520 nm to estimate the degree of non-enzymatic haemoglobin glycosylation. In phosphate buffer 0.01 M, pH 7.4, 2% glucose, 0.6% haemoglobin and 0.2% sodium azide solutions were produced. The combination described above was supplemented with 1 mL of each concentration of plant extract. The combination was kept at room temperature in the dark for 72 h. Colorimetric measurements were performed at 520 nm to determine the level of glycosylation of haemoglobin, and α-tocopherol (Trolox) was used as a reference medication in this experiment. The percentage of inhibition was estimated in accordance with a previously published technique [31]. All the tests were done in triplicate to ensure accuracy.

#### 2.6.4. Effect of SMFFs on Glucose Uptake by Yeast Cells

Commercial baker’s yeast was cleaned by repeated centrifugation (3000× *g*; 5 min) in distilled water until the supernatant fluids were clear. A 10% (*v/v*) suspension was made in distilled water, according to the procedure of Yeast cells. The extracts were then mixed with 1 mL of glucose solution (5, 10, and 25 mM) and incubated at 37 °C for 10 min. The reaction was initiated by adding 100 μL of yeast suspension, vortexing, and incubating for an additional 60 min at 37 °C. The tubes were centrifuged (2500× *g*, 5 min) after 60 min, and the glucose content of the supernatant was determined. Metformin was used as a reference medication for calculating the percentage increase in glucose absorption by yeast cells. Each test was conducted in triplicate [32].

### 2.7. Statistical Analysis

Dunnet’s ‘t’ test using One-way ANOVA was tested using GraphPad Prism version 8.0 for Windows and statistical methods to ensure a *p* < 0.05 was statistically significant.

### 2.8. Molecular Docking

The Glide XP docking procedure and the Schrodinger Maestro GUI were used to dock three main phytoconstituents of *S. mangifera* (β-amyrin, β-sitosterol and oleanolic acid) onto the active site of pancreatic α-amylase (PDB ID: 3BAJ, Resolution:2.10) to gain insight into their mechanisms of action. It was shown that β-amyrin, β-sitosterol and oleanolic acid had a high affinity for pancreatic α-amylase. Our docking strategy was validated by the co-crystallized ligand Acarbose, which demonstrated a binding posture comparable to that of the crystal structure [33]. When combined with α-amylase in their respective bound forms, β-amyrin, β-sitosterol and oleanolic, and acarbose showed high similarity. Hydrophobic couplings with β-amyrin, β-sitosterol and oleanolic acid were mediated by a group of residues that included Leu165A, Trp59A, and Trp62A. These residues are believed to be essential for pancreatic alpha production. alpha-amylase association [34].

### 2.9. Molecular Dynamic (MD) Simulation

GROMACS 2018.1 package systems were used to run the MD simulations for the top docked poses of the standard molecule, acarbose, and a-sitosterol complexes with pancreatic alpha-amylase (PDB ID: 3BAJ) using the OPLS-AA all-atom force field and the TIP3P water model, as described earlier [34]. In summary, topological parameters for simulated ligands were generated using the SwissParam web service (https://www.swissparam.ch/ (accessed on 10 February 2022) [35]. A cubic box with at least 1 nm separation from the protein-ligand complex was used to solvate and neutralize the complexes. The counter ions used were 0.15 M of (Na + Cl) as counter ions. A steepest descent algorithm approach was then used to reduce the energy consumption of the complexes. The lenience was 1000 kJ/mol/nm of maximum step size of 0.01 nm. The Linear Constraint Solver method was used to create bond length constraints (LINCS). Using the particle mesh Ewald (PME) approach, electrostatic calculations were performed. Equilibration was carried out for 100 ps utilizing “canonical ensembles NVT” and NPT (isobaric–isomerical ensemble). After then, a 20-ns production run was completed. Using GROMACS 2018.1 packages, key MD parameters such as root-mean square deviation (RMSD), root mean square fluctuations (RMSF), number of hydrogen-bond (HB), and radius of gyration (Rg) were investigated.

### 2.10. Physicochemical and ADMET Properties of S. mangifera Phytoconstituents

Molinspiration is web-based software used to predict drug-likeness properties of selected compounds. These parameters allow checking the drug absorptivity of selected compounds across the cell membrane and the potential of hydrogen bonding with their respective targets. The AdmetSAR ADMET online tool (http://www.admetexp.org (accessed on 10 February 2022) was used to determine physicochemical variables and ADME profile such as molecular hydrogen bond acceptor (HBA), hydrogen bond donor (HBD), molecular weight (MW), topological polar surface area (TPSA), rotatable bond count (RB), and octanol/water partition coefficient (LogP). In admetSAR, canonical smiles were uploaded of all the compounds, and results were obtained within a few minutes. This online tool implements the latest and most comprehensive manually curated data for miscellaneous compounds associated with known ADMET profiles.

## 3. Result

### 3.1. Quantitative HPLC Analysis of β-Sitosterol

**Figure 1:**
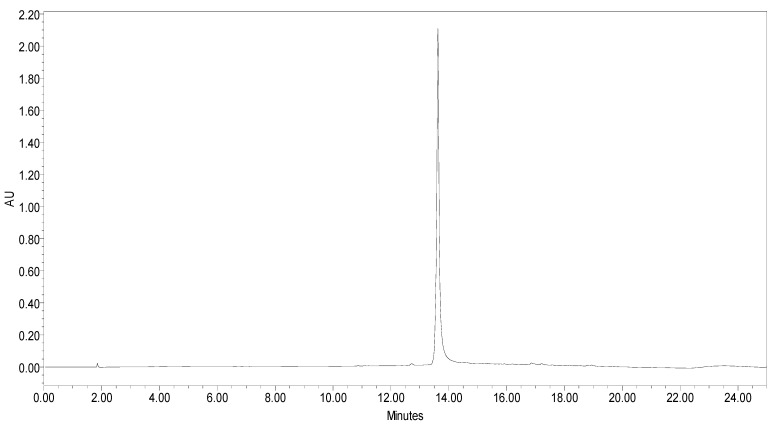
HPLC peak of standard β-sitosterol.

**Figure 2:**
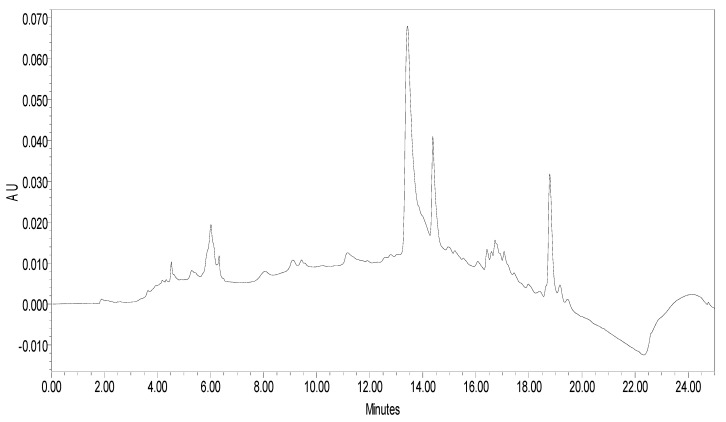
HPLC peak of β-sitosterol of *S. mangifera* fruit ethanolic fraction visualized at Rt 13.806.

β-sitosterol in extract was analysed using RP-HPLC using photo diode array (PDA) detector. RP-C18 column was used as stationary phase. The compound was detected at 212 nm. The linear regression calibration curves plotted between the peak area vs. concentration were linear for β-sitosterol, with good linear relationships (r^2^ = 0.98). Well-separated peak of β-sitosterol was visualized at Rt 13.806. HPLC peak of β-sitosterol of standard and sample are shown in Figure 1 and Figure 2. The percentage of β-sitosterol was found to be 1.21% ± 0.17% of total weight of extract (*w/w*).

### 3.2. Effect of SMFEF on ABTS Antioxidant Activity

**Figure 3:**
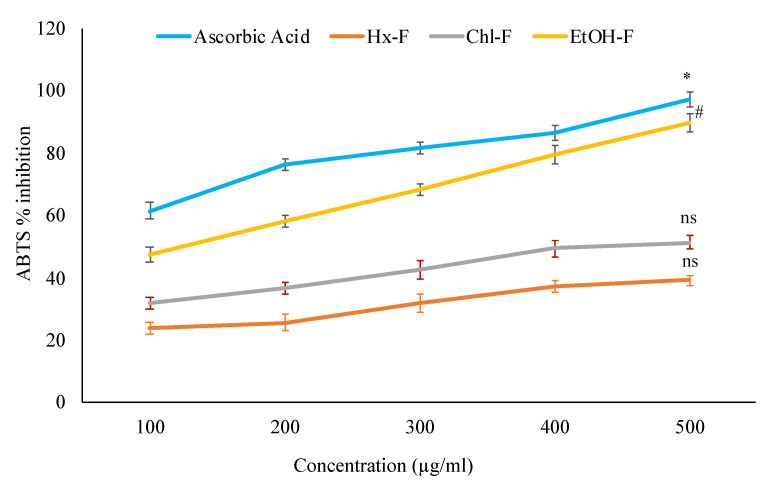
ABTS antioxidant activity of *S. mangifera* fruit fractions. Data are expressed as means ± SEM (*n* = 3), with a significance test for comparison with ascorbic acid using ANOVA followed by Dunnet’s ‘t’ test. * *p* < 0.01, # *p* < 0.05 and ^ns^ *p* > 0.05 = non-significant.

The present study examined various fractions of *S. mangifera* fruit extract for the ABTS radical scavenging activity. Standard ascorbic acid concentrations are used to compare different fractions scavenging effects. When compared with ascorbic acid (97.31 ± 3.67%), the ethanolic fraction (89.71 ± 2.73%) showed significant (*p* < 0.05) scavenging activity, but hexane (39.21 ± 3.29%) and chloroform (51.27 ± 2.79%) showed nonsignificant (*p* > 0.05) scavenging activity (Figure 3).

### 3.3. Effect of SMFEF on Percentage Inhibition of Lipid Peroxidation

**Figure 4:**
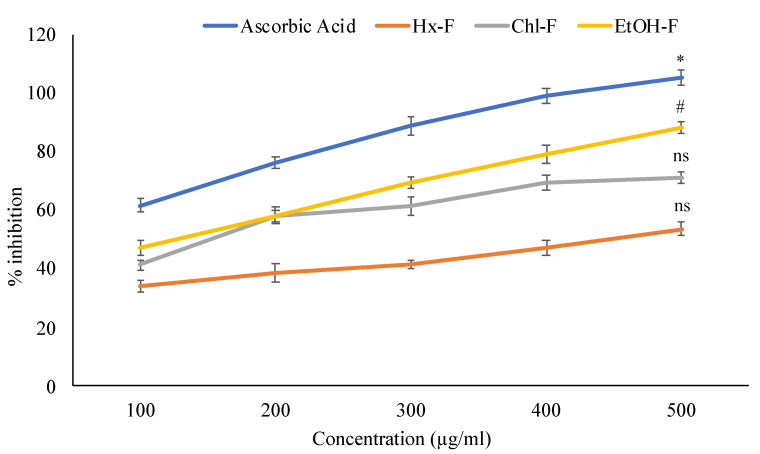
Percentage inhibition of *S. mangifera* fruit extract fractions on lipid peroxidation. Data are expressed as means ± SEM (*n* = 3), with a significance test for comparison with ascorbic acid using ANOVA followed by Dunnet’s ‘*t*’ test. * *p* < 0.01, # *p* < 0.05 and ^ns^ *p* > 0.05 = non-significant.

Concentration-dependent suppression of lipid peroxidation by *S.mangifera* fruit extract fractions was observed. The ethanolic fraction (88.26 ± 2.17%) showed maximum LPO activity at 500 µg/mL concentrations. In contrast, the other fraction, like hexane (53.69 ± 2.17%) chloroform (71.09 ± 1.94%), respectively, were showed less LPO activity when compared with standard ascorbic acid (105.21 ± 2.63%) shown in Figure 4.

### 3.4. Effect of SMFFs on Alpha-Amylase Inhibitory Activity

**Figure 5:**
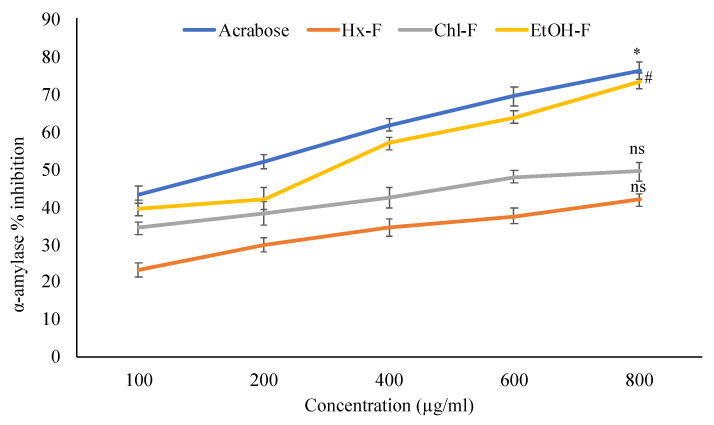
Percentage inhibition of *S. mangifera* fruit extract fractions (SMFEF) on α-amylase (*p* < 0.05). Data are expressed as means ± SEM (*n* = 3), with a significance test for comparison with acarbose using ANOVA followed by Dunnet’s ‘*t*’ test. * *p* < 0.01, # *p* < 0.05 and ^ns^ *p* > 0.05 = non-significant.

The maximum *in-vitro* α-amylase inhibitory study in an ethanolic fraction of *S. mangifera* fruit extract is shown in Figure 5. The percentage inhibitory activity alpha-amylase at 100, 200, 400, 600, and 800 μg/mL concentration of different fractions of extracts showed in a concentration-dependent manner. The ethanolic fraction showed significant (*p* < 0.05) alpha-amylase % inhibitory activity (73.42 ± 2.01%) at 800 μg/mL whereas the other fractions like hexane (41.83 ± 1.57%), chloroform (49.36 ± 2.39%) respectively, showed nonsignificant (*p* > 0.05) when compared with acarbose (76.21 ± 2.18%).

### 3.5. Effect of SMFEF on α-Glucosidase Inhibitory Activity

**Figure 6:**
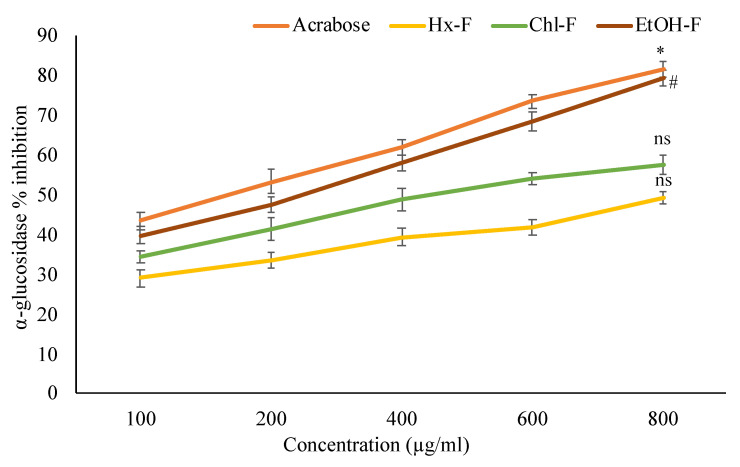
Percentage inhibition of *S. mangifera* fruit extract fractions (SMFEF) on α-glucosidase. Data are expressed as means ± SEM (*n* = 3), with a significance test for comparison with acarbose using ANOVA followed by Dunnet’s ‘*t*’ test. * *p* < 0.01, # *p* < 0.05 and ^ns^ *p* > 0.05 = non-significant.

Figure 6 revealed that the % inhibitory activity of *S. mangifera* fruit extract fractions showed concentration-dependent. The maximum % inhibitory alpha-glucosidase activity of *S. mangifera* fruit showed significantly (*p* < 0.05) in ethanolic fraction (79.23 ± 1.98%). In contrast, the other fractions like hexane (48.92 ± 1.57%) and chloroform (57.29 ± 2.39%) showed nonsignificant activity of % inhibition at 800 µg/mL when compared with standard acarbose (81.26 ± 2.01%) at the same concentration.

### 3.6. Effect of SMFEF on Percentage Inhibition of Glycosylated Hb

**Figure 7:**
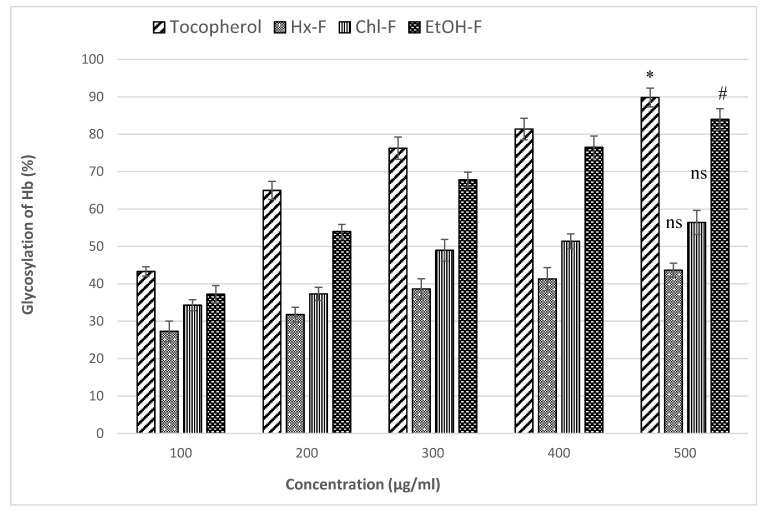
Percentage inhibition of non-enzymatic glycosylation of haemoglobin by *S. mangifera* fruit fractions (SMFEF). Data are expressed as means ± SEM (*n* = 3), with a significance test for comparison with Tocopherol using ANOVA followed by Dunnet’s ‘*t*’ test. * *p* < 0.01, # *p* < 0.05 and ^ns^ *p* > 0.05 = non-significant.

Figure 7 showed the percentage inhibition for the glycosylated Hb of *S. mangifera* fruit extract fractions at different concentrations like 100, 200, 300, 400, and 500 µg/mL. In this study, tocopherol was used as a standard same as the above concentrations. The maximum glycosylated Hb percentage inhibitory activity was significant (*p* < 0.05) shown in ethanolic fraction (83.97 ± 2.88%) at 500 µg/mL. In comparison, the hexane (43.64 ± 1.85%) and chloroform (56.39 ± 3.21%) fraction was showed nonsignificant (*p* > 0.05) glycosylated Hb percentage inhibitory activity when compared with tocopherol (89.92 ± 2.53%) at the same concentration.

### 3.7. Effect of SMFEF on Glucose Uptake by Yeast Cell

**Figure 8:**
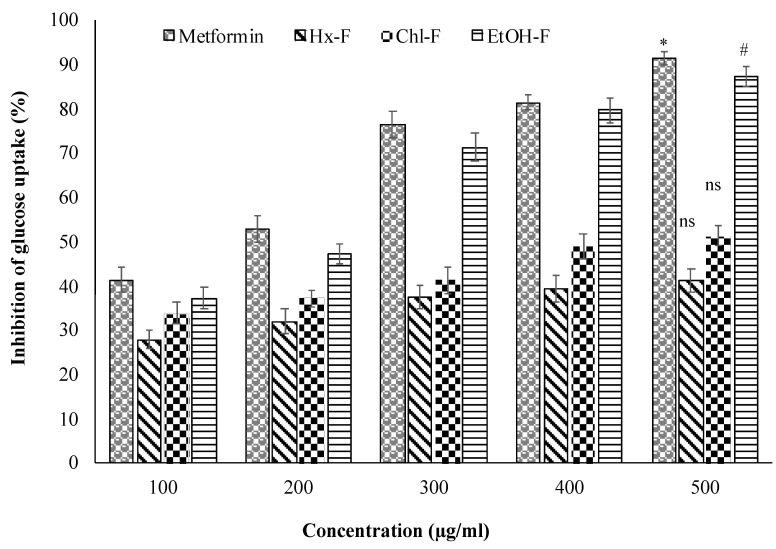
Inhibition of glucose uptake by yeast cell of *S. mangifera* fruit fractions (SMFEF). Data are expressed as means ± SEM (*n* = 3), with a significance test for comparison with Metformin using ANOVA followed by Dunnet’s ‘*t*’ test. * *p* < 0.01, # *p* < 0.05 and ^ns^ *p* > 0.05 = non-significant.

The ethanolic fraction of *S. mangifera* fruit promotes glucose uptake by the yeast cell across the plasma membrane. The glucose uptake of the ethanolic fraction by the yeast cell showed significant (*p* < 0.05) at 500 µg/mL when compared with metformin (91.37 ± 1.59%), whereas the other fraction did not show the uptake of glucose by the yeast cell at same 500 µg/mL concentration showed in Figure 8.

### 3.8. Molecular Docking

**Figure 9:**
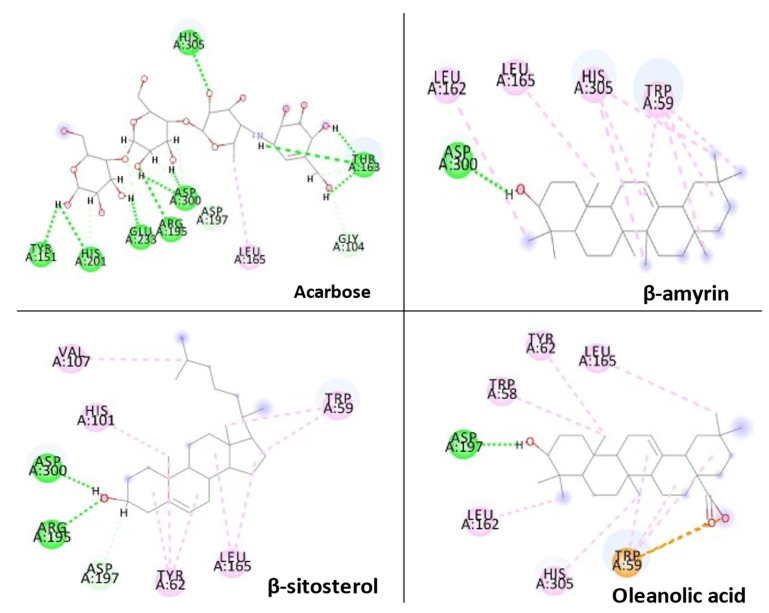
2D representation of docked conformation of α-amylase with ligands obtained after Glide XP docking.

**Figure 10:**
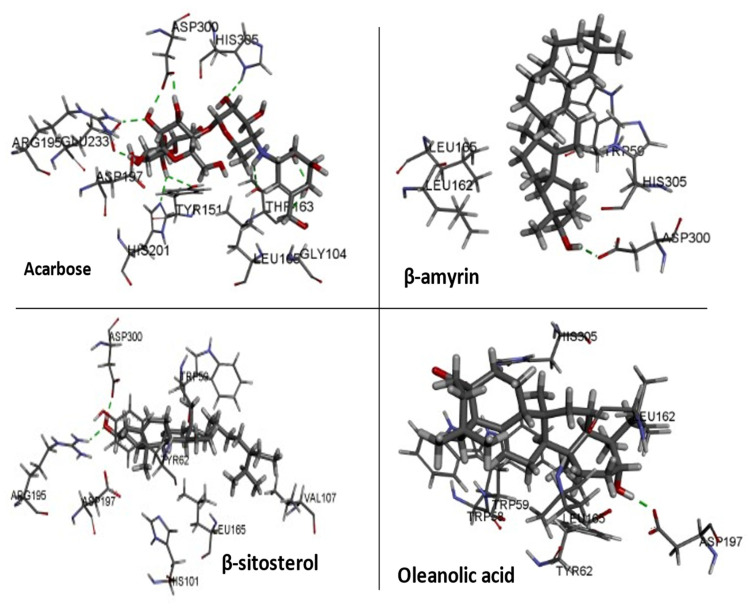
3D representation of docked conformation of alpha-amylase with ligands obtained after glide XP docking.

Three essential components were docked onto the active site of pancreatic α-amylase (PBD ID:3BAJ, Resolution:2.10), utilizing the Glide XP docking methodology and the Schrodinger Maestro GUI, to gain insight into their mechanism of action. Oleanolic acid, beta-sitosterol, and beta amyrin showed a strong affinity for pancreatic α-amylase. Our docking methodology was supported by the result of co-crystallized ligand Acarbose, which exhibited a similar binding posture to the crystal structure [36]. In addition, high similarities were shown by β-amyrin, β-sitosterol, oleanolic acid, and acarbose when coupled with α-amylase in their respective bound states. The hydrophobic interactions with β-amyrin, β-sitosterol, and oleanolic acid were mediated by a set of residues, including Leu165A, Trp59A, and Trp62A. These residues are reported to be important for pancreatic alpha-amylase binding [33]. The Leu162A Glide XP score and interacting binding residues are summarized in Table 1. Figure 9 and Figure 10 shows 2D and 3D depictions of β-amyrin, β-sitosterol, oleanolic acid, and acarbose binding at the interface of pancreatic α-amylase chain A.

### 3.9. Molecular Dynamic (MD) Simulation

**Figure 11:**
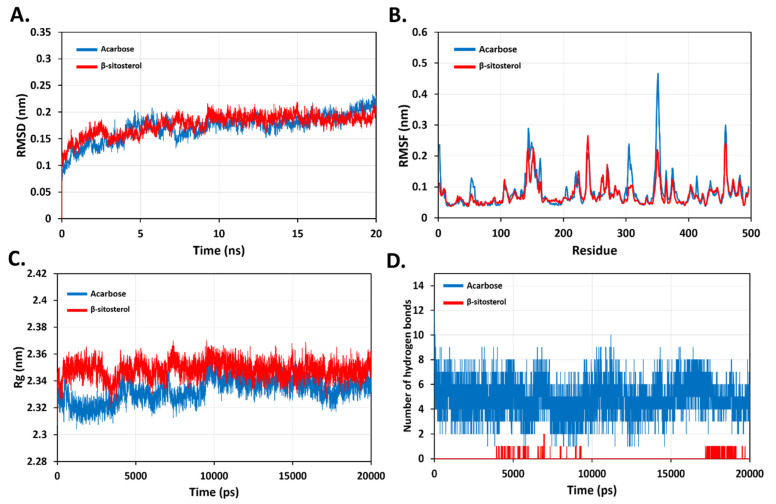
Dynamic behaviour and stability of ligands-protein complexes during molecular dynamics simulations. (**A**) Backbone RMSD for complexes. (**B**) Backbone RMSF for complexes. (**C**) Backbone Rg for complexes. (**D**) Number of formed hydrogen bonds.

To predict the dynamic behaviour and stability of β-sitosterol in the active site of pancreatic alpha-amylase, MD simulation was performed at 20 ns. The conformation and dynamic changes observed during simulation for pancreatic alpha-amylase in complex with β-sitosterol or acarbose are discussed herein. The calculated parameters including: RMSD, RMSF, Rg and hydrogen bonds of backbone atoms for β-sitosterol- pancreatic alpha-amylase complex were in reference to acarbose-pancreatic alpha-amylase complex as a standard complex (Figure 11). The average RMSD values for acarbose-pancreatic alpha-amylase and β-sitosterol-pancreatic alpha-amylase complexes were 0.17 (±0.03) nm and 0.18 (±0.02) nm, respectively. Thus, there was no observe any apparent differences between the ligand and reference RMSD indicating overall stability (Figure 11A). Also, the calculated RMSF values for both complexes have not crossed 2 nm for all atoms. The RMSF values were 0.10 (±0.05) nm and 0.19 (±0.04) nm for acarbose-pancreatic alpha-amylase and β-sitosterol-pancreatic alpha-amylase complexes, respectively. We observed less fluctuation in some residues such as Asn350, Gly351 and Asn352 that are located in the loop region away from the active site due to the binding of β-sitosterol (Figure 11B). Moreover, the analysis of radius of gyration of both complexes found no deviating suggested that both complexes were steady and compact during simulation time (Figure 11C). Next, hydrogen bonds between acarbose, β-sitosterol and the amino acids within the active site of pancreatic alpha-amylase were analysed. The standard ligand, acarbose, exhibited the highest interactions over time with up to 12 -10 hydrogen bonds, β-sitosterol was involved in around 2 hydrogen bonds with pancreatic alpha-amylase (Figure 11D). Overall, β-sitosterol could form a stable complex since it was confined in the active binding pocket of pancreatic alpha-amylase during the simulated period.

### 3.10. Pharmacokinetic Analysis via Molinspiron and ADMETSAR Webserver

In the current study, to screen out the potential pharmacokinetic properties of the main compounds of *S. mangifera* fruit was evaluated via Molinspiron and ADMETSAR webserver. β-amyrin, β-sitosterol, and oleanolic acid have been found to qualify all the rules having good permeability. Hence, it can be used as an effective drug, but its stability has to be verified with molecular simulation studies. The bioactivity score of β-Amyrin, β-Sitosterol, and oleanolic acid has greater than the acarbose, which concluded that β-amyrin, β-sitosterol, and oleanolic acid has good absorption, metabolism, and distribution. A higher bioactivity score also suggests that β-amyrin, β-sitosterol, and oleanolic acid can be potent inhibitors for nuclear receptor, protease inhibitors, and GPCRs (Table 2).

## 4. Discussion

*S. mangifera* fruits were extracted using a different polarity of solvents, including hexane, chloroform, and ethanol. The ABTS approach is quite practical and efficient for determining the scavenging activity of specified phytochemicals in plant extract fractions in a rapid, stable, and sensitive manner [28]. Compared to other scavenging tests, the ABTS technique may be utilized in organic and inorganic solvent systems. This approach provides a more accurate assessment of lipophilic and hydrophilic free radical scavenging activity [37]. The *S. mangifera* fruit ethanolic fraction (EtOH-F) has superior inhibitory efficacy compared to the hexane (Hx-F) and chloroform fractions (Chl-F), suggesting that the antioxidants in the hexane and chloroform fractions have a low ability to scavenge free radicals.

During a free radical-initiated oxidative chain reaction, lipid peroxidation occurs when each successive lipid molecular is oxidized to the highest degree feasible. This chain reaction usually stops when the substrate runs out. Alternatively, joining two radicals to generate nonradical products or reacting with antioxidants offer easily donatable hydrogen for peroxyl radical abstraction. This test may be performed enzymatically (Fe/NADPH) or nonenzymatic (Fe/ascorbic acid). As we employed egg yolk like a substrate, it is possible to conclude that *S. mangifera* can oxidize nonenzymatically. Our investigation discovered that SMFEF exhibited superior inhibitory action compared to other tested fractions in the reducing power experiment. The color of the test solution varies from yellow to different colors of green and blue based on the extract’s reducing ability. Some previous researchers have reported a clear association between antioxidant activity and the reducing capacity of specific plant extracts [38]. All extracts’ reducing power improves with increasing concentration, which is closely associated with their antioxidant activity. Therefore, these reducers must account for the antioxidant effects of extracts. Lipid peroxidation is a natural event that causes peroxidative loss at unsaturated lipids, resulting in lipid breakdown and membrane instability. Peroxidized lipids have been implicated in the pathophysiology of several illnesses and may act as a molecular basis of cell harm during pathological situations [39]. As a sign of oxidative stress, lipid peroxidation is often assessed in terms of its catabolite malondialdehyde (MDA) [40].

Diabetes is defined by abnormally high blood sugar levels, resulting in life-threatening consequences. It is characterized by elevated blood sugar levels, resulting in significant problems. Thus, the objective of diabetes treatment is to retain near-normal glycaemic control. Regrettably, there is no therapy or medication available in contemporary medicine that can effectively manage diabetes without causing adverse effects from insulin and oral hypoglycemic drugs. As a result, medicinal plants with antidiabetic effects may be helpful in the development of safer, more cost-effective antidiabetic medications. The anti-diabetic effect of *Spondias mangifera* fruit extract fractions were investigated in this study. α-Glucosidase is the enzyme responsible for the last step in the breakdown of carbs. It is found on the brush-border cell surface of the intestinal epithelium. α-Glucosidase inhibitors may slow the absorption of dietary carbs in the small intestine and decrease postprandial hyperglycemia, which might be a promising strategy for developing antidiabetic medicines. This is primarily utilized as a successful pharmaceutical treatment for hyperglycemia associated with type 2 diabetes in its early stages. A significant carbohydrate hydrolysis enzyme required for converting disaccharides and oligosaccharides to glucose is alpha (α)-glucosidase (GAA). This allows the small intestine to ingest the generated monosaccharides, resulting in an increase in blood glucose. GAA has been identified as the most important enzyme in the prevention and treatment of Type 2 diabetes. AGIs can help people with diabetes to maintain their blood glucose levels regular by slowing down the digestion of carbohydrates and reducing the absorption of monosaccharides. Consequently, screening AGIs is critical in preventing and treating type 2 diabetes (T2D). Currently, attempts are being made to screen AGIs derived from either organic substances or natural sources. As a result, screening for naturally occurring active chemicals with little adverse effects in natural goods as AGIs has gained increasing interest [41].

Antioxidative systems in the human body, including enzymatic and non-enzymatic mechanisms, reduce the creation of reactive oxygen species, which are linked to a wide range of degenerative disorders, such as diabetes. When blood glucose levels rise, it binds to haemoglobin, causing reactive oxygen species to develop [42]. *S. mangifera* fruit fractions strongly reduced haemoglobin glycosylation, as shown by increased haemoglobin concentrations that are shown by the formation of the glucose-haemoglobin complex, which increases free haemoglobin. The ethanolic fraction of *S. mangifera* fruit (EtOH-F) inhibited glycosylation more effectively than the chloroform or hexane extracts. The dose-dependent percentage reduction of glycosylation was observed. Controlling the glucose concentration in the blood of a diabetic patient may help avoid the disease’s numerous consequences. The ability of mammalian species to maintain a stable plasma glucose content over an extended period of time under a range of dietary patterns is one of the most critical and finely controlled processes identified [43].

Alpha-amylase, one of the intestinal digestive enzymes, is essential for carbohydrate digestion. *S. mangifera* has strong anti-diabetic action, as evidenced by in vitro α-amylase inhibitory tests (Figure 5). A dose-dependent relationship was seen for the percentage inhibition at doses of 100, 200, 400, 600, and 800 g/mL of crude plant extracts. The % inhibition of ethanolic fraction at 800 µg/mL was 73.42 ± 2.01%, chloroform extract was 49.36 ± 2.39%, and hexane was substantially less at 41.83 ± 1.57%. The alpha-amylase hydrolyses large alpha-linked polysaccharides like starch and glycogen to produce maltose and glucose. Our research discovered that compared to the standard acarbose 76.21 ± 2.18%, the ethanolic extract of *S. mangifera* inhibited alpha-amylase activity significantly. Skeletal muscles consume glucose due to an increase of functional glucose transporter molecules in the cell membrane. In response to increased insulin production in the blood, leptocytes and myocytes control glucose transporting molecules, resulting in hypoglycaemia [44].

The current study’s in vitro experiments demonstrated that the ethanolic extract had a favourable anti-diabetic effect. Figure 8 depicts the rate of glucose transfer through the cell membrane in the yeast cell system. Glucose transport in yeast (*Saccharomyces cerevisiae*) happens through facilitated diffusion. The glucose absorption of yeast cells was observed to increase dose-dependently upon treatment with these plant extracts. The *S. mangifera* fruit ethanolic fraction (EtOH-F) demonstrated considerably increased reactivity at all glucose concentrations, with the greatest increase seen at 500 g/mL glucose. Additionally, the results suggested that *S. mangifera* was highly effective in enhancing yeast cell glucose absorption when compared to the standard. Additionally, yeast cells may have a distinct glucose absorption mechanism than other eukaryotic or human body cells. Numerous factors, such as the glucose content within the cells or the subsequent glucose metabolism, might impact glucose absorption by yeast cells. For example, if the majority of the internal sugar is rapidly converted to other metabolites, the inner glucose level decreases, favouring glucose absorption into the cell. Similarly, glucose absorption by yeast cells in the presence of *S. mangifera* fruit extract fractions might result from enhanced diffusion and increased glucose metabolism.

Furthermore, when combined with α-amylase in their respective bound states in molecular docking studies, β-amyrin, β-sitosterol, oleanolic acid, and acarbose showed substantial similarity. Docking interaction of β-sitosterol compound (−5.68) showed the highest binding affinity. The dynamic behaviour and stability of β-sitosterol from MD simulation revealed the average RMSD values for acarbose-pancreatic alpha-amylase and β-sitosterol-pancreatic alpha-amylase complexes 0.17 (±0.03) nm and 0.18 (±0.02) nm, respectively. Thus, there was no observe any apparent differences between the ligand and reference RMSD indicating overall stability. The medication’s Drug Likeness is assessed by predicting the Absorption, Distribution, Metabolism, Excretion, and Toxicity (ADMET) features, which showed the pharmacokinetics of the selected compounds. The goal of computing ADMET profiles is to provide an early forecast of a compound’s in vivo performance so that its potential to become a drug may be assessed. According to Lipinski’s rule of five, it is governed by a complex balance of many molecular qualities and structural aspects, which impact the function of a component in a living organism. No more than one of the rules should be broken by a good drug contender [45].

β-sitosterol is the most common phytosterol and has shown a variety of biological effects. The RP-HPLC analysis of EtOH-F of *S. mangifera* showed the percentage of β-sitosterol 1.21% ± 0.17% of total weight of extract (*w/w*). It will undoubtedly be fascinating to investigate the activities of natural extracts (including *S. mangifera* fruit extract fractions) that may aid in improved glucose absorption by muscle cells and adipose tissues. The extract efficiently binds glucose and transfers it through the cell membrane for additional metabolism. This research yields novel biological and chemical data for the *S. mangifera*, and it holds the promise of exciting discoveries and possibility of further research.

## 5. Conclusions and Future Perspective

*S. mangifera* is a widely recognized plant with various phytoconstituents, such as oleanolic acid, β amyrin, and sitosterol. *S. mangifera* fruits from India’s tropical region are high in bioactive chemicals that have been shown to have health advantages. It may also be utilized as a food ingredient due to its antioxidant content. Therefore, this study studied antioxidant and antidiabetic properties of different fractions of *S. mangifera* fruit extract from Indian geographical origin via in vitro experimental assays and *silico* docking simulation studies. In addition HPLC analysis of EtOH-F was performed for the presence of β-sitosterol. In vitro, antioxidant activities were assessed using the ABTS and lipid peroxidation assay, while in vitro antidiabetic effects were assessed using enzyme inhibition assays of alpha-amylase, alpha-glucosidase, glycosylated Hb percentage inhibitory activity, and uptake of glucose by the yeast cell was examined. Docking experiments revealed that β-amyrin, β-sitosterol, and oleanolic acid had a strong binding affinity for human lysosomal a-glucosidase and human pancreatic a-amylase enzymes, exhibiting advantageous binding modes, stable protein-ligand complexes, and well-defined protein-ligand interactions. Docking interaction of β-sitosterol compound (−5.68) showed the highest binding affinity. The ADMET prediction research revealed that the main plant chemicals had admirable qualities to be used for oral medication. In vitro and *silico* studies showed that the *S. mangifera* fruit ethanolic fraction (EtOH-F) has high antioxidant capabilities and antidiabetic potential. Therefore, it may play a key role in drug development. Additional validation of in vitro and in silico investigations using in vivo assays is critical for establishing the antidiabetic efficacy of *S. mangifera* fruits.

## Figures and Tables

**Table 1:** Summary of selected constituents and reference compound (Acarbose); Chemical structures, Glide XP docking scores, hydrogen bond interactions and close contact residues.

Ligands	Chemical Structure	Binding Energy Score (Kcal/mol)	Interaction (H-Bond)	Interaction (Hydrophobic)
Acarbose	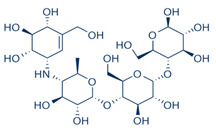	−14.90	His A:305, Thr A:163, Asp A: 300, Ard A:195, Glu A:233, His A: 201, Tyr A:151, Gly A:104	Leu A: 165
β-amyrin	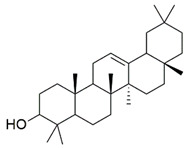	−4.38	Asp A: 300	Leu A:162, Leu A:165, His A:305, TrpA:59
β-sitosterol	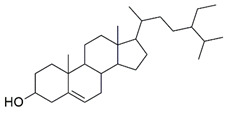	−5.68	Asp A:300, Asp A:197, Arg A:195	Val A:107, His A:101, TrpA:59, Leu A:165, Tyr A:62
Oleanolic acid	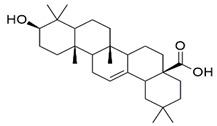	−4.18	Asp A: 197	TrpA:58, TrpA:62, Leu A:165, HisA:305, Leu A:162

**Table 2:** ADME analyses of the selected bioactive compounds of *S. mangifera* fruit.

Parameters	β-Amyrin	β-Sitosterol	Oleanolic Acid
Molecular weight	426.72 g/mol	414.71 g/mol	456.70 g/mol
No. of heavy atoms	31	30	33
No. Aromatic atoms	0	0	0
Fraction Csp3	0.93	0.93	0.90
No. rotatable bonds	0	6	1
No. H-bond acceptors	1	1	1
No. H-bond donors	1	1	2
Mol. Refractivity	134.88	133.23	136.65
TPSA	20.23 Å^2^	20.23 Å^2^	57.53 Å^2^
iLOGP	4.74	4.79	3.89
XLOGP	39.1	59.3	47.49
WLOGP	8.17	8.02	7.23
MLOGP	6.92	6.73	5.82
Silicos-IT Log P	6.92	7.04	5.85
Log P	7.18	7.19	6.06
ESOL Log S	−8.25	−7.90	−7.32
Solubility (mg/mL)	2.40 × 10^−6^	5.23 × 10^−6^	2.16 × 10^−5^
Solubility (mol/L)	5.62 × 10^−9^	1.26 × 10^−8^	4.74 × 10^−8^
Class	Poorly soluble	Poorly soluble	Poorly soluble
Log S (Ali class)	−9.47	−9.67	−8.53
Silicos-IT	−7.16	−6.19	−6.12
Silicos-IT Class	Poorly soluble	Poorly soluble	Poorly soluble
GI absorption	Low	Low	Low
BBB permeant	No	No	No
Pgp substrate	No	No	No
CYP1A2 inhibitor	No inhibitor	No inhibitor	No inhibitor
CYP2C19 inhibitor	No inhibitor	No inhibitor	No inhibitor
CYP2C9 inhibitor	No inhibitor	No inhibitor	No inhibitor
CYP2D6 inhibitor	No inhibitor	No inhibitor	No inhibitor
CYP3A4 inhibitor	No inhibitor	No inhibitor	No inhibitor
Log K_p_ (skin permeation)	−2.41 cm/s	−2.20 cm/s	−3.77 cm/s
Lipinski No of violation	1	1	1
GHOS No of violation	3	3	3
Veber No of violation	Yes	Yes	Yes
Egan No. violation	1	1	1
Muegge No violation	2	2	1
Bioavailability Score	0.55	0.55	0.85
PAINS	0	0	0
BRENK	1	1	1
Lead likeness No. violation	2	2	2
Synthetic accessibility	6.04	6.30	6.08

## Data Availability

Not applicable.
